# Systematic evaluation of a targeted gene capture sequencing panel for molecular diagnosis of retinitis pigmentosa

**DOI:** 10.1371/journal.pone.0185237

**Published:** 2018-04-11

**Authors:** Hui Huang, Yanhua Chen, Huishuang Chen, Yuanyuan Ma, Pei-Wen Chiang, Jing Zhong, Xuyang Liu, Asan, Jing Wu, Yan Su, Xin Li, Jianlian Deng, Yingping Huang, Xinxin Zhang, Yang Li, Ning Fan, Ying Wang, Lihui Tang, Jinting Shen, Meiyan Chen, Xiuqing Zhang, Deng Te, Santasree Banerjee, Hui Liu, Ming Qi, Xin Yi

**Affiliations:** 1 BGI-Shenzhen, Shenzhen, China; 2 School of Bioscience and Bioengineering, South China University of Technology, Guangzhou, China; 3 Casey Eye Institute Molecular Diagnostic Laboratory, Portland, Oregon, United States of America; 4 Shenzhen Eye Hospital, Jinan University, Shenzhen, China; 5 BGI-Tianjin, BGI-Shenzhen, Tianjin, China; 6 Key Laboratory of Optoelectronic Devices and Systems of Guangdong Province, Shenzhen University, Shenzhen, China; 7 Shenzhen Key Laboratory of Genomics, Shenzhen, China; 8 The Guangdong Enterprise Key Laboratory of Human Disease Genomics, Shenzhen, China; 9 Nanshan Maternity & Child Healthcare Hospital of Shenzhen, Shenzhen, China; 10 Maternity and Child Health Hospital of Anhui Province, The Maternal and Child Health Clinical College, Anhui Medical University, Hefei, China; 11 School of Basic Medical Sciences, Zhejiang University, Hangzhou, China; 12 Functional Genomics Center, Department of Pathology & Laboratory Medicine, University of Rochester Medical Center, West Henrietta, New York, United States of America; University of Florida, UNITED STATES

## Abstract

**Background:**

Inherited eye diseases are major causes of vision loss in both children and adults. Inherited eye diseases are characterized by clinical variability and pronounced genetic heterogeneity. Genetic testing may provide an accurate diagnosis for ophthalmic genetic disorders and allow gene therapy for specific diseases.

**Methods:**

A targeted gene capture panel was designed to capture exons of 283 inherited eye disease genes including 58 known causative retinitis pigmentosa (RP) genes. 180 samples were tested with this panel, 68 were previously tested by Sanger sequencing. Systematic evaluation of our method and comprehensive molecular diagnosis were carried on 99 RP patients.

**Results:**

96.85% targeted regions were covered by at least 20 folds, the accuracy of variants detection was 99.994%. In 4 of the 68 samples previously tested by Sanger sequencing, mutations of other diseases not consisting with the clinical diagnosis were detected by next-generation sequencing (NGS) not Sanger. Among the 99 RP patients, 64 (64.6%) were detected with pathogenic mutations, while in 3 patients, it was inconsistent between molecular diagnosis and their initial clinical diagnosis. After revisiting, one patient’s clinical diagnosis was reclassified. In addition, 3 patients were found carrying large deletions.

**Conclusions:**

We have systematically evaluated our method and compared it with Sanger sequencing, and have identified a large number of novel mutations in a cohort of 99 RP patients. The results showed a sufficient accuracy of our method and suggested the importance of molecular diagnosis in clinical diagnosis.

## Introduction

Inherited ophthalmic disorders are a large group of clinically and genetically heterogeneous retinal diseases that constitute a major cause of blindness in children and adults [1]. This heterogeneity includes genetic, allelic as well as clinical heterogeneity [2, 3]. Hereditary retinal diseases, which consist of a group of blinding diseases, such as Cone-Rod Dystrophy (CRD) and RP, are the most common ophthalmologic genetic disorders. Although most of the monogenic eye diseases remain untreatable at current stage [4]. Advances in genetic studies make it possible to reveal more than 200 disease-causing genes associated with more than 30 retinal diseases (RetNet: https://sph.uth.edu/retnet/sum-dis.htm), paving the way to accurate diagnoses, prognoses and effective genetic counseling, reducing the risk of disease recurrence in families at risk as well as improving the mechanism-specific care for these diseases [5,6].

However, there is still substantial gap in clinical gene testing of monogenic eye diseases [7]. Many monogenic eye diseases, such as RP, CRD and Leber congenital amaurosis (LCA), display a very high degree of genetic heterogeneity (each includes 20 to 60 known diseases causing genes). In additional, because of the lack of clear gene inheritance pattern in many patients and the absence of distinctive phenotypes among diseases caused by different genes, parallel sequencing of nearly all known genes were required in both research and clinical genetic screening in contexts of these diseases. Genetic eye disease can be inherited as autosomal recessive (ar) or autosomal dominant (ad), X-linked (XL), or mitochondrial traits. Nevertheless, the majority of cases are sporadic. Identifying the genetic cause of the patients’ disease is crucial for genetic counseling of patients and families, and is a prerequisite for any form of genotype-based therapies. However, the enormous genetic heterogeneity in inherited ophthalmic disorders makes attempts to identify causative mutations a challenging task [8]. Recent progresses in NGS and target-enrichment technologies, which enable simultaneous rapid sequencing and analysis of the sequences of hundreds of genes at high accuracy and several orders of magnitude reduced cost, provide new opportunity for bridging the gaps in gene testing of monogenic eye diseases [9, 10]. Actually, targeted gene capture NGS methods applied in research context of RP and LCA gene testing raise the possibility of being used as a routine diagnostic tool in clinical contexts [11, 12]. Till now, ten more labs are providing prenatal testing service or carrier testing service through sequence analysis of the entire coding region. Emory Genetics Laboratory developed eye disorders panel include 143 genes related to 141 kinds of eye diseases (http://www.ncbi.nlm.nih.gov/).

Here, we present the development of a systematic monogenic eye diseases gene testing panel of 283 genes by coupling NGS and solution-phase-hybridization based target-enrichment method. After evaluation of key technical parameters, such as reproducibility, the sequencing depth for variants calling and accuracy of variants detection, we sequenced a cohort of 64 patients tested by Sanger sequencing previously, to compare the clinical sensitivity of these two methods, and tested the performance of the panel assay in molecular diagnosis by screening 99 unselected Chinese RP patients. Our results demonstrated that the gene testing panel is a cost-effective and high-throughput method that could be applied in both research and clinically molecular diagnosis of genetic eye diseases.

## Materials and methods

### Samples

The DNA samples of 180 individuals, 163 are patient samples (with extra 12 family member samples), 68 (64patients + 4family members) of which were de-identified samples from the Casey Eye Institute, USA. The IRB approval number is IRB00008083 and the study title is “Development of new diagnostic tests”. These 163 samples include 20 samples with previously identified causative variants, 44 samples with incomplete or no information of causative variants in previous Sanger sequencing-based gene testing, and 99 RP patients without any gene testing information (Table 1). The YH DNA sample (C004 and C005) was provided by BGI-Shenzhen. The Ethics Committee of BGI has approved this study, and the IRB approval number is BGI-IRB 14002. Informed consents obtained from patients were approved by the respective institutional review boards or research ethics board.

**Table 1:** Sample information.

tested by sanger sequencing (68)			without any gene testing (107)		control
positive	negative	one mutation	RP	Two RP family	
**20 patients & 4 family members**	20	24	97	2 patients & 8 family members	5

### Disease gene collections and targeted capture probes design

283 genes for 146 monogenic eye diseases, which include 58 known RP related genes (S1 File), were collected from database (OMIM: http://www.ncbi.nlm.nih.gov/omim/, and RetNet: https://sph.uth.edu/Retnet/sum-dis.htm). The RefSeq entries of these genes were also given in S2 File. Customized oligonucletide probes were designed to capture the exonic sequences and 30bp around exons by NimbleGen (Roche) oligonucletide probe design system.

### Targeted sequencing library preparation and sequencing

Targeted sequencing libraries were prepared as follows: 1μg genomic DNA was sonicated to 20~300 bp sized fragments, followed by end-repair, A-tailing, and Illumina adaptors ligation, then 4 cycles pre-capture PCR and sample barcode indexing; And then, the indexed PCR product of 20–30 samples were pooled. Then, targeted capture was performed by hybridizing with capture probes, and followed by 15 cycles of PCR amplification and validation the library products for sequencing. DNA sequencing was done on Illumina HiSeq2000 sequencers to generate 90 bps of paired-end reads and 8 bps of the sample barcode.

### Data filtering and analysis

Image analysis and base calling were finished by the build-in Pipeline of Illumina. Indexed primers were used for the data fidelity surveillance. Only reads that matched the adapter and primer indexed sequences with no more than 3nt mismatches were identified as valid reads. Sequencing statistics of coverage, depth and coverage depth were listed in Table 2. The reference was obtained from the NCBI, version GRCh37 (hg19). Sequence alignment was performed using Burrows Wheeler Aligner (BWA) Multi-Vision software package [13]. SNVs were called by SOAPsnp [14] and small InDels (<20bp) were identified using the Samtools (Tools for alignments in the SAM format) Version: 0.1.18, http://samtools.sourceforge.net/.

**Table 2:** Detailed primer sequence for real-time PCR.

Gene	Primer	Primer sequence	Size/bp	Tm/°C
**CACNA2D4**	Exon 17_Forward	CCCACTCCACTTCGGAGAGAT	75	60.2
	Exon 17_Reverse	TCCAGTACAGAGAGGGGAAGAAAC	75	60.2
	Exon 20_Forward	TACCGACCCACCTTCTTCCA	85	59.8
	Exon 20_Reverse	CCACAGTTTGGGGGTGGTG	85	59.8
	Exon 26_Forward	GCTGTCCTCTTGTCCACGGT	78	60.5
	Exon 26_Reverse	TTCCCCAGAAAGTGCGGGTG	78	60.5
**CRX**	Exon 1_Forward	CGAGTTCCAGGCCATGACAA	148	59.8
	Exon 1_Reverse	CTTCAGAAAGGAGGGACGGG	148	59.8
	Exon 2_Forward	CCCGAAGATCATGATGGCGT	90	59.6
	Exon 2_Reverse	TGCATCAGATCCACACTGGG	90	59.6
	Exon 4_Forward	TGACCGCTGAAGTACACCAC	132	60.7
	Exon 4_Reverse	TGTTCAAGGGCACGGTGATT	132	60.7
**TULP1**	Exon 9_Forward	GTGCGGAGAGAACAGAGAGG	118	60.5
	Exon 9_Reverse	CTCCCCAGAGCCTCCTAACT	118	60.5
	Exon 11_Forward	CCTCCTCGGGACAGATTGGT	77	59.5
	Exon 11_Reverse	GCTGGCAGGAAACGAAAACG	77	59.5
	Exon 13_Forward	ATGGGGACCCTCTCGTTCTC	86	59.8
	Exon 13_Reverse	GAAACCAACGTGCTGGGCTT	86	59.8

### Copy number variations detection

The screening method of copy number variations (CNV) we use was previously described by Wei XM et al. [15]. The cut-off value was built on the precondition that suggests significant depth correlation (r>0.7) at the sequencing exons among each sample. Then, z-score was calculated according to the depth of each capture region. Particularly, z-score (>2.58) was selected as the cut-off value since it filters out> 99% normal samples for bilateral tailed region. Regions with absolute z-score (>2.58) were defined as deletion (<-2.58) or duplication (>2.58) regions.

$$z=\frac{\bar{X}-\mu }{\sigma }$$

In this formula: $\bar{X}={\text{Nom}}_{\text{exon}}=\frac{\text{mean}\;\text{depth}\;\text{of}\;\text{certain}\;\text{exon}}{\text{mean}\;\text{depth}\;\text{of}\;\text{all}\;\text{target}\;\text{region}\;\;\text{in}\;\text{the}\;\text{same}\;\text{sample}}$; $\mu =\frac{\sum {\;\text{Nom}}_{\text{exon}}}{\text{N}}$; It means average mean depth of one certain exon in all samples; σ: Standard deviation of one certain exon in all samples from the same batch.

### Quantitative real-time PCR (qPCR)

In order to validate the CNV results of our method, Quantitative real-time PCR (qPCR) analysis was performed. The exon 17, 20, 26 of gene *CACNA2D4*, exon 1, 2, 4 of gene *CRX*, exon 9, 11, 13 of gene *TULP1* were measured by qPCR using an ABI 7900HT Real-time PCR system (Life Technologies, Carlsbad, CA, USA) and HS qPCR Master Mix, according to the manufacturer’s instructions. The primers used for amplifying these exons were listed in Table 3.

**Table 3:** Sequencing statistics of coverage, depth and coverage depth.

index	Average
**reads_sequenced(Mb)**	15.2
**reads_mapped_to_hg19(Mb)**	97.10%
**reads_mapped_to_target_region**	67.36%
**reads_mapped_to_flank_region**	77.21%
**data_mapped_to_target_region**	44.42%
**data_mapped_to_flank_region**	54.81%
**sequencing_depth_of_target region (fold)**	400
**sequencing_depth_of_flank region (fold)**	295
**1X_coverage_depth_of_target region**	98.23%
**4X_coverage_depth_of_target region**	97.85%
**20X_coverage_depth_of_target region**	96.85%
**1X_coverage_depth_of_flank region**	98.24%
**4X_coverage_depth_of_flank region**	97.73%
**20X_coverage_depth_of_flank region**	94.66%

Flank region: 200bp around target region

The PCR procedure was initiated with a thorough denaturation step of 95°C for 10 min followed by amplification cycles. The amplification cycle condition was 95°C for 10 s, annealing (annealing temperature was specific for each pair of primers) for 15 s and 72°C for 30 s, for a total of 45 cycles. The DNA copy number level in affected samples were compared with the level in control samples from normal individual.

### Mutation interpretation procedure

In order to identify disease causing mutations we applied the following four-step procedure. The first stage is to find out the mutations that could lead to protein coding change, which are, stop (nonsense), missense variants, exonic small insertions/deletions (InDels), especially frameshift, InDels and variants at potential canonical splice sites (±10bp of exons). Next, we will quote the allelic frequency in three databases, i.e. 1000 human genome dataset, dbSNP database. A variant having allelic frequency greater than 0.01 in any one of the databases will be filter out, as the diseases we studied here are very rare disease. In order to exclude the genetic polymorphism variants predominantly found in Chinese population, we sequenced the 283 genes of 200 Chinese normal person to build our internal control database. And thirdly, we used 5softwares (SIFT, PolyPhen2, Mutation taster, FATHMM, PhyloP score) in dbNSFP to predict novel missense variants, the variants which are predicted to be Damage or possible Damage (PhyloP score>0 will be treat as “Damage”) in at least two software were reserved. Finally, if no mutations could be found from the first three stages, we will check the CNV (copy number variants) for the candidate pathogenic genes.

## Results

### Systematic evaluation of the method

In order to evaluate the method, we performed targeted gene capture sequencing of the protein coding regions and 30 bp immediately adjacent sequences of 283 genes on 83 samples, including one cohort of 68 samples (64 patients + 4 family members) that have been tested by Sanger sequencing in Casey Eye Institute, USA, and 2 RP families (10 members in total), 5 unaffected healthy samples. We indexed each of the 83 samples individually and performed 4 targeted capture experiments with each pooling of 20–30 samples.

### Coverage and depth analysis of 283 monogenic eye diseases genes

On an average, we generated 15.2 Mb high-quality reads for each sample, with 67.36% of which mapped onto targeted regions, corresponding to an average coverage of 400 folds on targeted regions. This sequencing depth results in at least 97.85% and 96.85% of each targeted region covered by at least 4 and 20 folds, respectively (Table 2). Among the total 4381 exons of 283 genes, only 54 exons from 35 genes were poorly covered (<50%) because of presumably high GC content or repetitive nature of the sequences and could be complemented by Sanger sequencing of PCR products.

**Fig 1:**
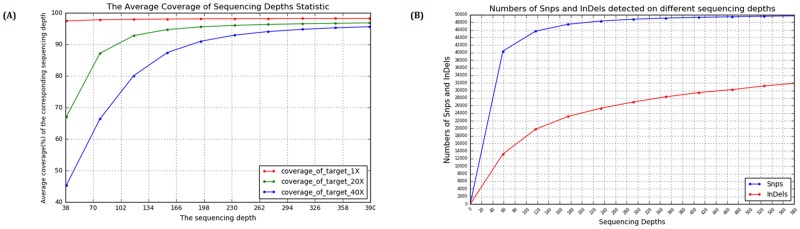
Analysis of coverage depth and variants detection. A. The coverage of target region with 1, 20, 40 folds with increasing sequencing depths. B. Total number of SNPs and InDels detected with increasing sequencing depths.

Moreover, with respect to determine the least sequencing depth required for reasonable targeted region coverage and variants detection, we randomly extracted subsets of reads with different average depths from the total mapped reads for each sample. At a sequencing depth between 200–250 folds, the coverage of targeted regions reached 97.5% with at least 1 read and 95.6% with at least 20 reads, both of which did not show any remarkable improvement with further increase in sequencing depth (Fig 1A). For the depth need for variants calling, the number of identified SNPs also saturated at a sequencing depth of >200 folds, a similar trend was also observed for the detection of Indels (Fig 1B). Thus, an average sequencing depth over 200 folds in samples for this study is adequate for a reasonable coverage of targeted regions and variant detection.

### Reproducibility and accuracy of variants detection

**Fig 2:**
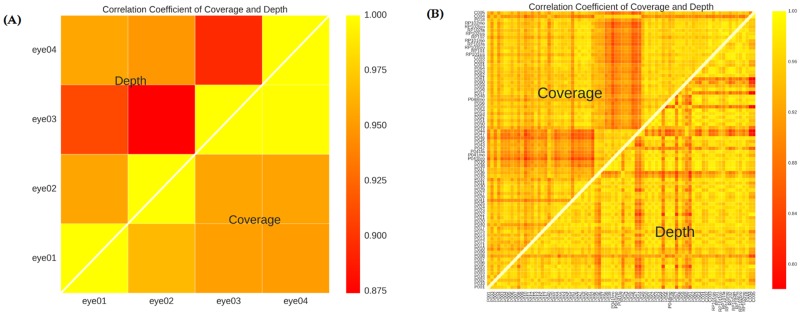
Correlation of coverage rate and sequencing depth on consensus targeted exons. The graph shows pair-wise Pearson correlation coefficients for both sequencing coverage (top-left triangle) and depth rate (bottom-right triangle) based on 4381 exons targeted by our eye chips. A. Correlation of sequencing coverage and depth rate on consensus targeted exons of the samples of the 4 targeted capture experiments. B. Correlation of sequencing coverage and depth rate on consensus targeted exons of 67 samples.

In order to assess the reproducibility of the targeted gene capture sequencing panel, we calculated the correlation coefficient of coverage rate and mean sequencing depth on target regions among samples for intra and inter of the 4 targeted capture experiments. Result of each batch showed high reproducibility (0.816 to 0.996 of correlation coefficient for coverage and 0.781 to 0.999 for depth). A very high and comparable level of correlations (coverage >0.816 and depth > 0.781) were observed for both intra- and inter-experiment measurements (Fig 2A and 2B), indicating the general reliability of the targeted genes. The relative high correlation coefficient for depth and coverage rate was expected since the sequencing depth was sufficiently high, and the coverage of most target regions has reached saturation, so that the random fluctuation was in a reasonable range. Hence, the targeted gene sequencing method has a high level of reproducibility that is acceptable in relative studies.

In order to assess the accuracy of variants detection, we also performed targeted sequencing for the YH (C005) sample, the genome of which has been deeply sequenced by whole genome sequencing for 50 folds. By the variant detection methods and according to the criteria described in *Materials and Methods*, we identified 911SNPs both in targeted sequencing and YH genome data for 1,505,712bp exon region of the 283 genes, respectively. Among those SNPs, there were 868 SNPs overlapped between two data sets, 43 SNPs specific to targeted sequencing and 43 SNPs specific to YH genome data. According to these results, both the maximum false positive rate (FP rate) and false negative rate (FN rate) are 4.7%. The sensitivity, specificity, precision and accuracy were calculated as follows:

Sensitivityortruepositiverate=TP/(TP+FN)=868/(868+43)=95.3%

Specificityortruenegativerate=TN/(FP+TN)=1504758/(43+1504758)=99.997%

Precisionorpositivepredictivevalue=TP/(TP+FP)=868/(868+43)=95.3%

Accuracy=(TP+TN)/(P+N)=(TP+TN)/(TP+FN+TN+FP)=(868+1504758)/(868+43+1504758+43)=99.994%

### Mutations identification for the clinical samples previously tested by Sanger sequencing

Furthermore, with respect to compare the identification of pathogenic mutations (clinical sensitivity) between our panel NGS method and Sanger sequencing, we applied this method to 68 clinical samples (64from patients + 4from family members) which were previously tested by Sanger sequencing. As a result, on average, 973 variants in exons of the 283 genes for every sample, including 520 SNVs and 453 InDels, were detected. And among 66,194 variants, 5,559(8.40%) were from intron, 29,881 (45.1%) were from UTR and 30,754 (46.5%) from CDS. Further annotation displayed that there were 13,531missense, 4,750 splice-sites, 23 nonsenses, 17,105 synomymous SNPs, and 95 coding Indels.

In our comparative experiment with Sanger sequencing, we successfully detected all the mutations from 20 patients which were identified by Sanger sequencing. For the rest of the 44 patients, only one mutation was detected in each of 24 patients with by Sanger sequencing, 20 patients received negative result by Sanger sequencing. The results of these 44 patients by NGS screening were consistent with the results of Sanger sequencing except for 4 patients (Table 4), it showed that pathogenic mutations revealed by NGS could cover the detection spectrum of Sanger sequencing. Because our NGS panel includes more eye disease genes which were not included in the previous Sanger RP gene list, the discrepant results of the 4 patients in Table 4 were mutations found in genes not sequenced by Sanger sequencing. For example, patient P007 was diagnosed autosomal recessive RP, we identified compound heterozygous mutations in *BBS2*: p.Arg275X and p.Pro134Arg, the nonsense mutation was found pathogenic and most likely has a significant effect on the function of the protein complexes [17–19]. The p.Pro134Arg mutation was novel and predicted probably damaging byPolyPhen-2 software (http://genetics.bwh.harvard.edu/pph2/). In patient P010, the two mutations in *BBS1* gene, c.1645G>T(p.Glu549X) and c.1169T>G(p.Met390Arg), have been reported in previous studies [20, 21]. It was well known that mutations in BBS (Bardet-Biedl syndrome) associate with gene induced syndromes characterized by the visual defect and other systemic symptoms like renal abnormalities. But it was also reported that ‘RP-like’ phenotypes without impairment in other organs was related to BBS genes in some cases [22, 23]. The patients P007 and P010 were diagnosed as arRP and arLCA, yet the pathogenic mutations were found in BBS related genes instead of RP or LCA associated genes. Similar situation was found in the RP patient P062 and LCA patient P064. In patient P062, compound heterozygous mutations of *CRB1*: p. Cys948Tyr/p.165_167delAspGlyIle were detected, both the mutations were reported pathogenic [24, 25]. Patient P064 revealed compound heterozygous mutations of *CNGB3*: c.1600_1601insTT/p.Gly567Glu, the insertion mutation results in frameshift mutation leads to premature termination of translation of CNGB3 transcript, and the missense mutation was a novel variation predicted pathogenic by PolyPhen-2.

**Table 4:** Inconsistent result between NGS and Sanger in four patients.

Sample number	Disease	Mutations			Sanger result
**P007**	arRP	BBS2	c.823C>T(p.Arg275*) (het)	**c.401C>G(p.Pro134Arg)(het)**	negative
**P010**	arLCA	BBS1	c.1645G>T(p.Glu549*)(het)	c.1169T>G(p. Met390Arg) (het)	negative
**P062**		CRB1	c.2843G>A(p.Cys948Tyr) (het)	c.493_501delGATGGAATT (het)	One mutation
**P064**	arLCA	CNGB3	**c.1600_1601insTT (het)**	**c.1700G>A(p.Gly567Glu) (het)**	negative

Variants shown in bold type are novel.

**Fig 3:**
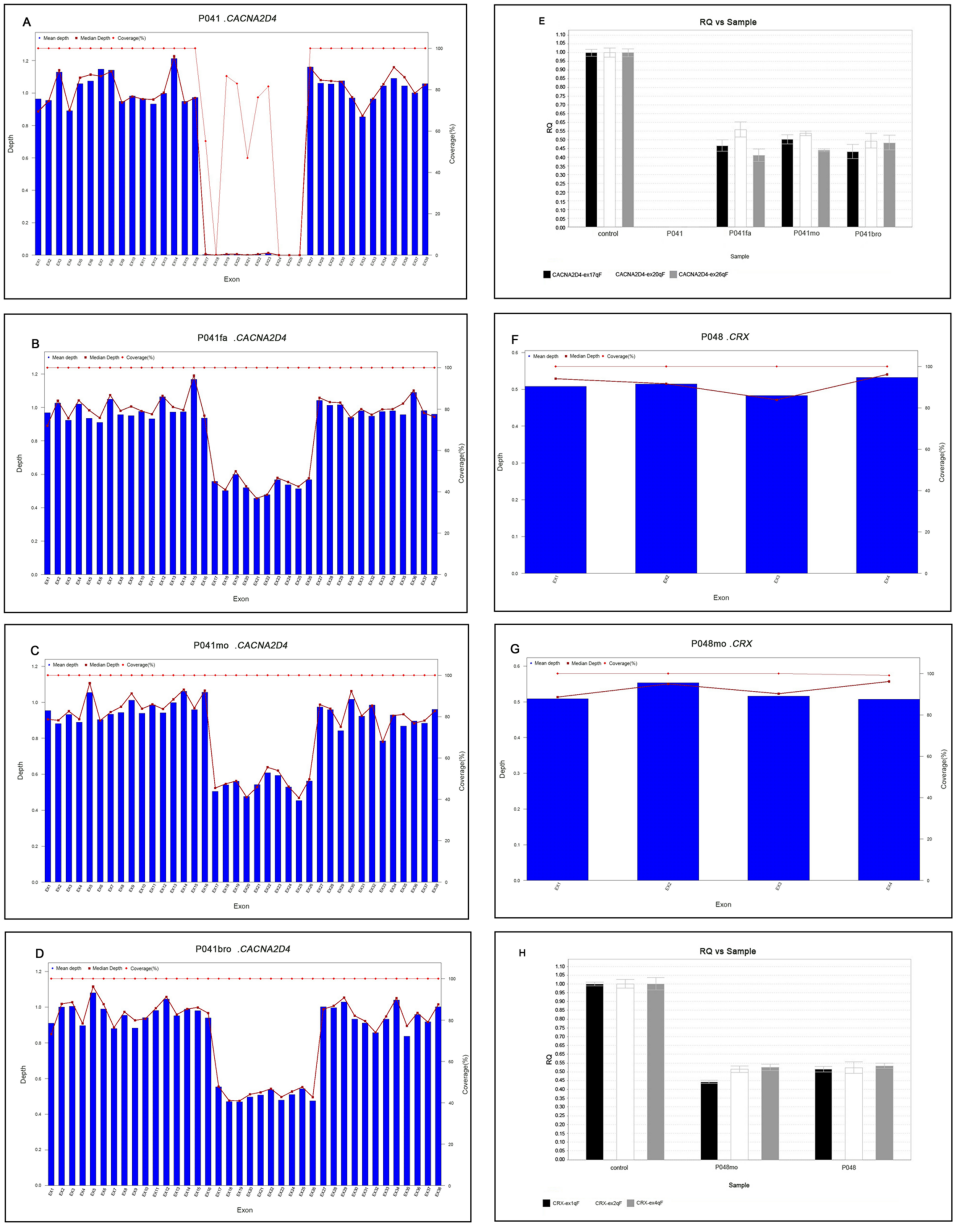
Large deletion in CACNA2D4 and CRX gene identified by analysis of the normalized sequencing depth, and confirmed by quantitative PCR. A. P041: Patient (proband) B. P041fa: father (carrier) C. P041mo: Mother (carrier) D. P041Bro: Brother (carrier) E. quantitative PCR result of P041 family F. P048: Patient (proband) G. P048mo: Mother (affected) H. quantitative PCR result of P048 family.

Other than SNPs and small Indels, our NGS-based study also determines copy-number variation (CNV). For instance, patient P041 was diagnosed with retinal CRD. Our results showed a homozygous deletion of EX.17_26 exons within *CACNA2D4* gene (Fig 3A) which was found related to retinal CRD in previous studies [26]. Meanwhile, we also found the patient’s family members—father, mother and brother, carried a heterozygous deletion of EX. 17_26 within *CACNA2D4* gene (Fig 3). The z-score of 17–26 exons were greater than 4.0 in patient P041, and almost all z-score were greater than 2.58 in his father, mother and brother. Consistently, the quantitative Real Time PCR (qPCR) result further validated the CNV of *CACNA2D4* gene in P041 family (Fig 3E). This deletion was once found in late onset bipolar disorder patients [27]. Similar situations were found in patient P048, he and his mother were found to carry a heterozygous deletion in the whole *CRX* gene, mutations in *CRX* are associated either with recessive LCA or with dominant CRD. (Fig 3).

### Molecular diagnosis of 99 RP samples

After the systematic evaluation of our panel, to test the significance of our method in molecular diagnosis, we performed the molecular diagnosis on 99 unselected Chinese RP patients, which also includes 6 Bietti Crystalline Corneoretinal Dystrophy (BCD) patients.

### Sequencing of 99 RP patients using the developed panel

Using the above mentioned panel, we performed the targeted gene capture NGS experiment on 99 unrelated Chinese patients with clinical diagnosis of RP, and then the bioinformatics analysis was performed (described in Materials and methods). An average of 322 folds sequencing depth was achieved, 68.7% reads were mapped to the target region, and 98.1%, 97.2% of bases in target region were covered by 4X, 20X respectively, indicating that sufficient sequencing depth and coverage was obtained to detect variants. A total of 93,242 SNPs and 8965 InDels were identified in 99 samples, and on average, 541.8 SNPs and 490.6 small InDels were identified for each sample, respectively. Since RP is a rare mendelian disease, the variants with a frequency <0.01 in 1000 genome database, dbSNP and HapMap were kept only. In addition, to filter out the polymorphic variants in Chinese population, the variants with a frequency <0.05 in our internal database (see part “mutation interpretation” in Methods) were kept only. As a result, 52.4 rare variants (SNPs + InDels), on average, were only left in each sample, there were about 19 rare variants left in protein coding region and potential splice site. Finally, we used a “dbNSFP” program that includes 5 prediction algorithms (SIFT [28], PolyPhen-2 [29], Mutation Taster [30], FATHMM [31], PhyloP score [32]) to predict the pathogenicity of novel missense variants. As the results of prediction algorithms were often contradictory; we just took the prediction results as a reference.

### Molecular diagnosis in 99 RP patients

**Fig 4:**
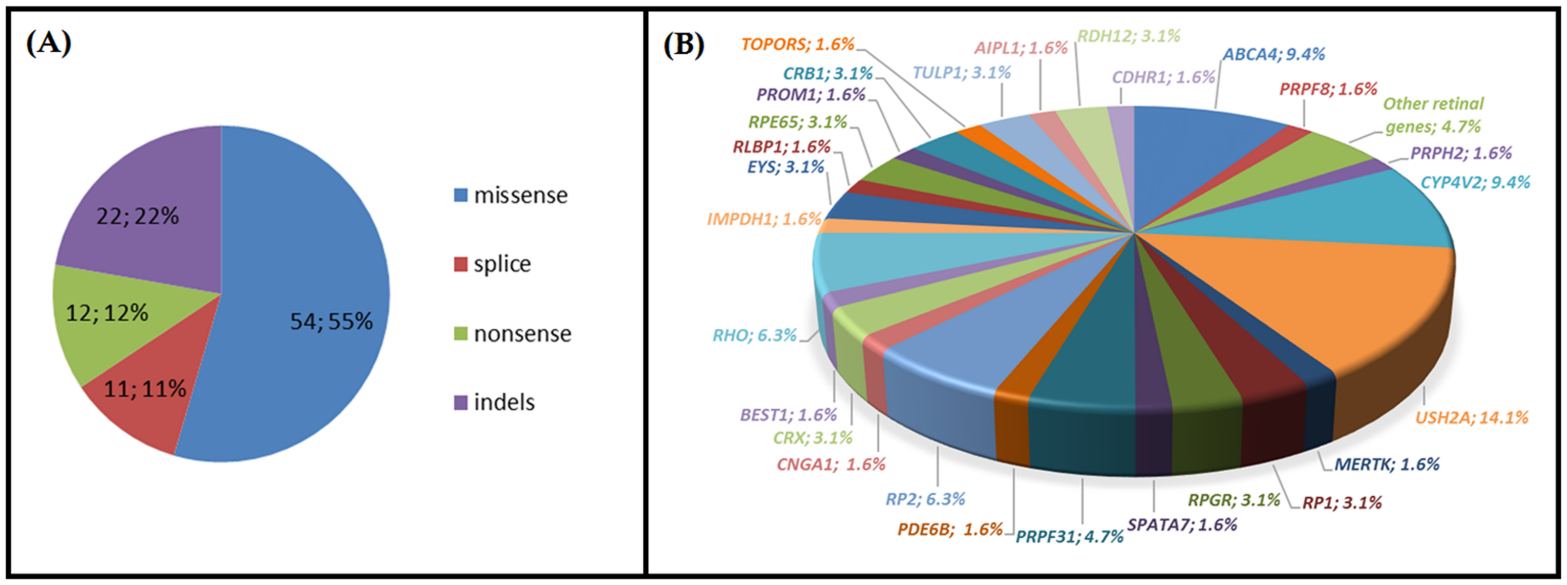
The spectrogram of disease-causing genes and mutations in 99 RP patients. The molecular diagnosis statistics of 99 RP patients: A. The percentage of different types of pathogenic mutations. B. The percentage of different types of pathogenic genes.

Following our procedures, we identified 99 mutations diagnosed in all 99 RP patients, all the pathogenic mutations were validated by Sanger sequencing (RP original 54 genes). As major components, missense mutations constitute 55% and the splice, nonsense and InDel mutations together are responsible for 35% of the total identified mutations respectively (Fig 4A). We detected mutations consistent with RP phenotype in 61 (16 autosomal dominant, 40 autosomal recessive and 5 X-linked) out of 99 cases, and there are also mutations in 3 cases explained other retinal diseases such as LCA and fundus albipunctatus. Thus our identification rate was 63.5% (61/96) for RP patients and 64.6% (64/99) for all patients (Fig 4B). Altogether, we identified 94 mutations in 27 different RP genes and 5 mutations in other 3 retinal diseases genes. Among them, 72 are novel mutations and 27 are previously reported mutations. The distributing of these 27 RP disease-causing genes identified in patients was neither equally nor partially to one or two genes. The most common gene is *USH2A* that accounted for 9 cases, while mutations in *ABCA4* and *CYP4V2* genes were identified in 6 cases respectively. Eventually, recurrent mutations in patients were rare, few patients carried the same mutations, but the c.802-8_c.810del/insGC mutation in *CYP4V2* was more frequent in BIETTI CRYSTALLINE CORNEORETINAL DYSTROPHY (BCD, OMIM #210370), due to the founder effect in Asian [33]. In order to understand the co-segregation of the mutations clearly, the phenotype segregation analysis was performed in 16 cases, segregation analysis turned out to be concord with the molecular diagnosis in all 16 cases.

Herein, in accordance with inheritance mode, after the pathogenic mutations were identified, the patients with potential RP-causing mutations were classified into 3 groups based on the confidence levels of different patients; patients detected with all reported mutations were defined as highest confidence group (Group. 1). Patients identified with at least one novel frameshift /nonsense mutations were categorized as middle confidence group (Group. 2). Patients carrying only novel missense/splice mutations were defined as lower confidence group (Group. 3). We identified 14 patients, 16 patients, and 31 patients in group 1, 2 and 3, respectively.

**Fig 5:**
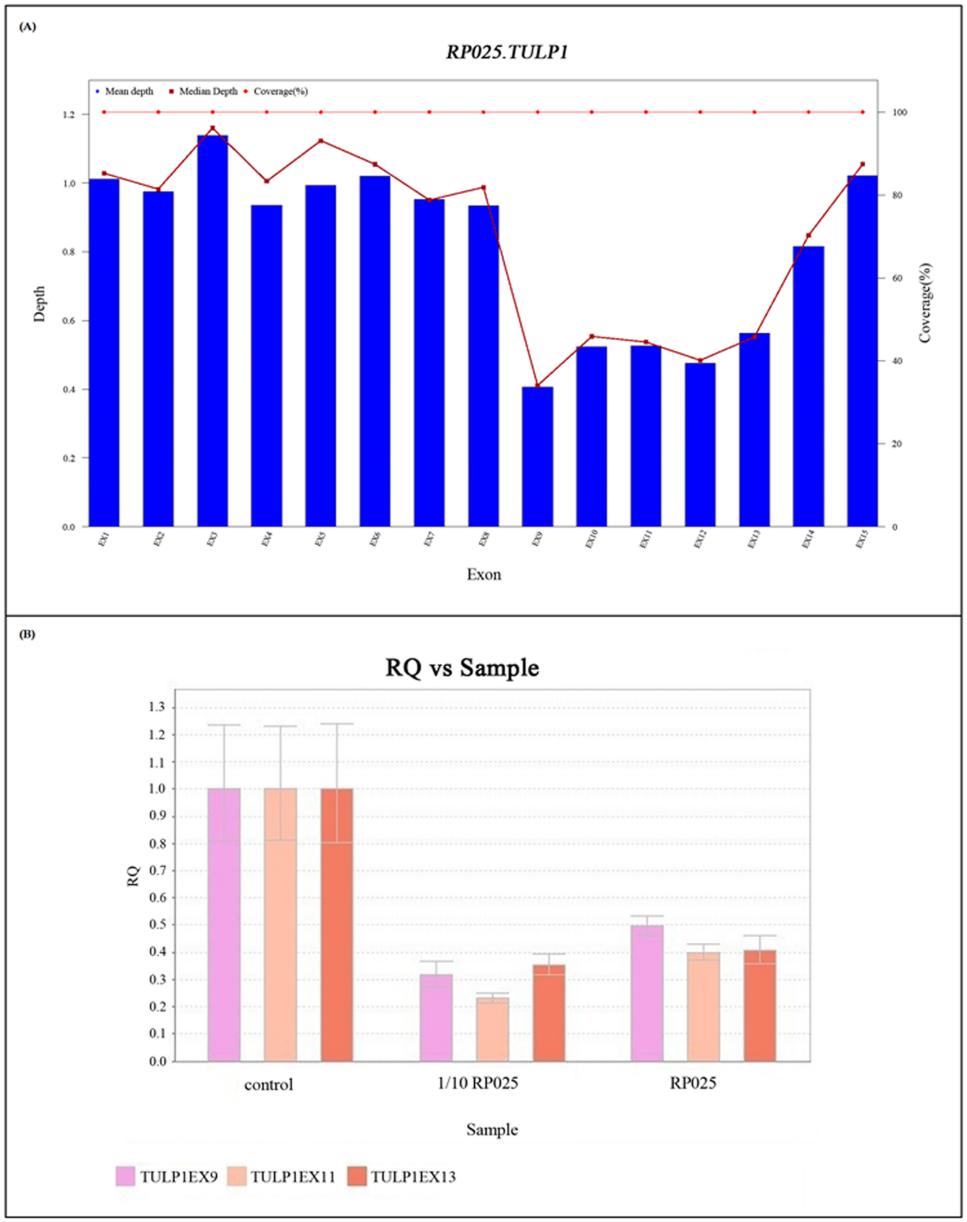
Large deletion in *TULP1* gene identified by analysis of the normalized sequencing depth, and confirmed by quantitative Real Time PCR (qPCR). A. Normalized sequencing depth of exons in *TULP1* gene in patient RP025; B. Quantitative Real-Time PCR (qPCR) result. 1/10 RP025 is a repeat for the quantitative PCR using 1/10 initial concentration of RP025 DNA.

Other than SNPs and small InDels, we also found a patient, YK13S0025, carried a heterozygous deletion of exon 9–13 within *TULP1* gene as well as a heterozygous variant c.349G>A (p.Glu117Lys) in *TULP1* gene. Subsequently, qPCR has been applied to this large deletion for validation (Fig 5). There is a distribution bias of *TULP1* pathogenic mutations which occurs in exons 10 to 15 [34, 35]. The deletion of exon 9–13 results in a loss of C terminus which contains the most conserved region among the *tub* family members and was assumed to be critical for *TULP1* function [36].

### Clinical revisiting of patients carrying mutations in non-RP-causing genes

Finally, among the 3 cases explained other retinal diseases, 2 patients carrying novel frameshift/ InDel mutations were defined with high confidence and 1 patient carrying novel missense/splice mutations were defined with low confidence (Table 5). We revisited patient RP023 andRP095.

**Table 5:** Three patients carrying mutations in other retinal diseases-causing gene.

Patient ID	Gene	Related disease	Type	Mutations
**RP095**	RDH5	Fundus albipunctatus; CSNB;CRD	Homozygous	c.928delCinsGAAG (p.Leu310delinsGluVal)
**RP001**	CEP290	LCA10; BBS14;Joubert syndrome 5; Meckel syndrome4;Senior-Loken syndrome 6;	Compound heterozygous	c.1666_1666delA
				(p.Ile556Phefs*17)
				c.5226 +5_8delGTAA
**RP023**	NMNAT1	LCA9	Compound heterozygous	c.-57 +7 T>G
				c.710G>A(p.Arg237His)

CRD, Cone-rod dystrophy; LCA, Leber congenital amaurosis; CSNB, Congenital Stationary Night Blindness;BBS, Bardet-Biedlsyndrome

Patient RP023 is a 33 years old man. He carried a novel splice-site mutation c.-57 +7T>G and a novel missense mutation p.Arg237His in LCA9 related gene, *NMNAT1* gene (Table 5) [37]. This patient showed night blindness and patchy losses of peripheral visual field since the age of 8 years. Visual acuity decrease gradually since the age of 12 followed by nystagmus, tunel vision, metamorphopsia and muscaevolitantes. His best corrected visual acuity (BCVA) was 20/200 and 20/50 in the right and left eye respectively. Fundus examination revealed waxy disc, obviously attenuated retina vascular. Significant pigmentary changes of salt and pepper or bone corpuscle type were noted. All these symptoms suggest that the clinical diagnosis is likely to be RP accompanied with cataract rather than LCA.

Patient RP095 is a 26 years old man. He carried a homozygous InDel mutation c.928delinsGAAG in *RDH5* gene. He exhibited symptoms as night blindness in childhood accompanied with myodystony in the left body occasionally. Scotopic ERG (rod response) after 30min dark adaption showed the a- and b-waves’s amplitudes reduced more than that of the condition of patient of 2 years earlier. Fudus examination disclosed periphery macula white starry dots, waxy disc, and obviously attenuated retina vascular without any significant pigmentary changes. Hence, the Clinical diagnosis is changed to fundus albipunctatus.

## Discussion

In this study, we developed and systematically evaluated a NGS based panel for molecular diagnosis of inherited ophthalmic disorders. The evaluation result demonstrates that our method has reached a significance in molecular diagnosis and a high standard of analysis parameters, clinical sensitivity comparing with Sanger sequencing.

99.994% accuracy of variant detection is achieved in this panel, and clinical sensitivity is not only as high as Sanger sequencing, but seems to show another advantage. Asan et al. did the correlation coefficient of coverage and depth analysis in their study, and the results of their coverage rate (0.65 to 0.78) was lower than mean depth (0.90 to 0.96) [16]. Contrast to our results, the lower correlation coefficient of coverage rate in their study may due to the 30 folds low sequencing depth, which made the random fluctuation wide. For example four patients were found to carry mutations in genes related to other genetic eye diseases which were not considered in Sanger sequencing (Table 4), novel mutations were in bold type in this table. The detection of mutations in these four patients may not be achieved if the screening was only performed on specific genes associated with one or several similar diseases, due to the variety of phenotype in some non-syndromic and especially syndromic diseases. For example, the clinical manifestations of LCA/RP and related retinal diseases may be various and overlapped both at early and late stages, which makes the discrimination of various retinal dystrophies difficult sometimes (Neveling et al., 2013) [38]. For example, patients who were diagnosed with RP/LCA may actually carry mutations in non-canonical LCA/RP genes. Hence, the clinical diagnosis should be refined by molecular diagnosis. Also, screening a larger set of genes related to ophthalmologic genetic diseases is essential, for the purpose of achieving a more accurate clinical diagnosis in these patients.

In addition, our method can detect large deletions. A homozygous 17^th^-26^th^ exons deletion in *CACNA2D4* and a heterozygous deletion of the whole *CRX* gene in two families (P041, P048 respectively) were identified by Casey Eye Institute and also by our method (Fig 3). RP025, one of the 99 RP patients was also found to carry a large heterozygous deletion in *TULP1* gene (Fig 5). In the past, people need to use two different methods to detect copy-number variants and SNVs, small InDels. Here, our pipeline can detect these 3 kinds of variants by one test. The algorithm of CNV detection is based on sequencing depth and Z-score module [15]. This pipeline can raise the molecular diagnosis rate and reduce the cost. In our opinion, it is the tendency to detect more genes and more kinds of variants by one test.

The molecular diagnosis rate of 63.5% was achieved for 96 Chinese RP patients using our method, while several recent studies using the NGS method for retinal diseases achieved a molecular diagnosis rate varying from 25–57% [38, 39]. Our panel is flexible in identifying multiple pathogenic genes or heterogeneous disorders associated mutations. It reduces the dependence of specific knowledge and skills in clinical diagnosis, and even also can provide evidence to modify clinical diagnosis. In all of the 99 patients, we found molecular diagnosis of three samples inconsistent with the initial clinical diagnosis, and then we revisited two patients, the clinical diagnosis of patient RP095 was reclassified from RP to fundus albipunctatus, while patient RP023 still presented a RP phenotype rather than LCA

The discrepancy in patient RP023 may be explained by the diversity of genotype-phenotype correlations, because it was reported that a lot of previously unsolved cases turned out to have mutations in genes relating to other retinal disease but not necessarily RP [40]. This explanation may also be suitable for the patient RP001. Patient RP001 carried a novel frameshift mutation c.1666delA and a novel splice site mutation c.5226+5_8delGTAA in *CEP290* gene, which is a frequent cause of LCA [41, 42]. This 26-year-old patient exhibited “RP-like” phenotypes for 14 years including night blindness, vision impairment and visual field constriction without defects in other organs. The above symptoms were not the most typical symptoms in LCA.

However, there are several possible reasons for undetected cases: (a) A few exons were poorly captured due to the difficulty in designing bait in repeat regions or the poor capture efficiency in GC-rich regions. Analyzing the coverage of 283 genes in all samples, 97.60% of genes were cover by ≥1x coverage for at least 90% of their coding bases, 97 genes doesn’t reach 100%, and 49 genes doesn’t reach 99%, while 4 genes were lower than 80% coverage. (b) We can identify the CNV, but deep intronic mutations and structural genomic variants were undetected. (c) Finally, some unsolved cases may be caused by new disease-causing genes while some may be caused by our limited understanding of the plethora of variants detected by NGS at present. Therefore, some variants could be overlooked by assuming they are non-pathogenic while others may be predicted pathogenic while indeed they are not.

The tremendous genetic and phenotypic heterogeneity of retinal diseases poses a major challenge for establishing a molecular diagnosis [43]. In the post-genomic era, NGS has revolutionized biological research and discovery. Thus, targeted gene capture is being used as a cost-effective alternative to WGS for investigating regions of interest when a prior knowledge of potentially causal loci is available [44].

In conclusion, we performed the systematic evaluation in our targeted gene capture sequencing panel, and have compared our method with Sanger sequencing. Our method showed a high performance, and we succeeded in identifying 64.6%pathogenic mutations for 99 unselected RP patients. Altogether 75 novel mutations were found. The results showed that our method is sufficiently accurate for molecular diagnosis, it also suggested a significance of molecular diagnosis in clinical diagnosis. Comprehensive genetic screening for eye diseases would allow genetics and clinicians to improve diagnosis and perform treatment trials using updated molecular diagnosis technologies [7]. Genetic screening will be an integral part of the care for hereditary eye disease patients, and the strategy used here will become a commonly used tool for the genetically heterogeneous eye disorders in the next few years.

## Conclusions

In summary, our study confirms the diagnostic value of NGS platforms in the identification of mutations in a heterogeneous disease like retinal disease. The advantage of WES to discover novel genes together with its reliable variant calling of coding regions and competitive prices, make it the technique of choice in the mutation screening of heterogeneous diseases. The aim of this study was to evaluate whether the target gene capture sequencing panel is appropriate for molecular diagnosis of genetic eye diseases. And we have systematically evaluated our method and compared it with Sanger sequencing. We have also identified a large number of novel mutations in a cohort of 99 RP patients. The experiments also showed some advantages:

Firstly, our method has a little higher clinical sensitivity than that of Sanger sequencing. Secondly, the 64.6%rate of molecular diagnosis suggested that our method was appropriate for molecular diagnosis and very helpful to confirm the clinical diagnosis. Third, our method can detect SNVs, small InDels and CNVs at one test, which is helpful to lower the cost and shorten the waiting time.

These results suggested that our method was sufficiently accurate for molecular diagnosis and suggested the importance of molecular diagnosis in clinical diagnosis.

## Supporting information

S1 File S1 File provides gene lists of 283 captured genes and 58 known RP disease-causing genes. (RAR)

S2 File This file contains the following sub-files: Figures A-C, Tables A-E and the references of the detected mutations in Tables A-E. Figure A shows the overall coverage of genes in the panel. Figures B and C show the genes that doesn’t reach 100% and 99%, respectively. Table A shows the variant numbers detected by NGS of 68 samples have previously tested by Sanger sequencing before. Table B shows the results of all 68 samples previously screened by Sanger sequencing. Table C shows the Z-score results for CNV detecting of family P041 and P048. Table D: Statistics of targeted NGS in 99 RP patients, shows the depth, coverage and variant numbers detected in 99 RP patients. Table E shows the mutations identified in 61 out of 99 RP patients.
(RAR)

## References

[1] Chiang JP, Trzupek K. The current status of molecular diagnosis of inherited retinal dystrophies. Curr Opin Ophthalmol. 2015 7; 26(5):346–51. doi: 10.1097/ICU.0000000000000185 26214332 10.1097/ICU.0000000000000185

[2] Weisschuh N, Mayer AK, Strom TM, Kohl S, Glöckle N, Schubach M, et al Mutation Detection in Patients with Retinal Dystrophies Using Targeted Next Generation Sequencing. PLoS One. 2016, 14; 11(1): e0145951 doi: 10.1371/journal.pone.0145951 26766544 10.1371/journal.pone.0145951 PMC4713063

[3] Lee K, Garg S. Navigating the current landscape of clinical genetic testing for inherited retinal dystrophies. Genet Med. 2015;17(4):245–52 doi: 10.1038/gim.2015.15 25790163 10.1038/gim.2015.15

[4] Young TL. Ophthalmic genetics/inherited eye disease. Curr Opin Ophthalmol 2003, 14:296–303. 14502058 10.1097/00055735-200310000-00011

[5] Glöckle N, Kohl S, Mohr J, Scheurenbrand T, Sprecher A, Weisschuh N, et al Panel-based next generation sequencing as a reliable and efficient technique to detect mutations in unselected patients with retinal dystrophies. Eur J Hum Genet. 2014, 22(1):99–104. doi: 10.1038/ejhg.2013.72 23591405 10.1038/ejhg.2013.72 PMC3865404

[6] Cideciyan AV, Swider M, Aleman TS, Tsybovsky Y, Schwartz SB, Windsor EA, et al ABCA4 disease progression and a proposed strategy for gene therapy. Hum Mol Genet 2009, 18:931–941. doi: 10.1093/hmg/ddn421 19074458 10.1093/hmg/ddn421 PMC2640207

[7] Bainbridge JW, Smith AJ, Barker SS, Robbie S, Henderson R, Balaggan K, et al Effect of gene therapy on visual function in Leber’s congenital amaurosis. N Engl J Med 2008, 358:2231–2239. doi: 10.1056/NEJMoa0802268 18441371 10.1056/NEJMoa0802268

[8] Drack AV, Lambert SR, Stone EM. From the laboratory to the clinic: molecular genetic testing in pediatric ophthalmology. American journal of ophthalmology 2010, 149:10–17. doi: 10.1016/j.ajo.2009.08.038 20103038 10.1016/j.ajo.2009.08.038 PMC2813223

[9] Bowne SJ, Sullivan LS, Koboldt DC, Ding L, Fulton R, Abbott RM, et al Identification of disease-causing mutations in autosomal dominant retinitis pigmentosa (adRP) using next-generation DNA sequencing. Investigative ophthalmology & visual science 2011, 52:494–503. 20861475 10.1167/iovs.10-6180 PMC3053293

[10] Audo I, Bujakowska KM, Leveillard T, Mohand-Said S, Lancelot ME, Germain A, et al Development and application of a next-generation-sequencing (NGS) approach to detect known and novel gene defects underlying retinal diseases. Orphanet J Rare Dis 2012, 7:8 doi: 10.1186/1750-1172-7-8 22277662 10.1186/1750-1172-7-8 PMC3352121

[11] Coppieters F, De Wilde B, Lefever S, De Meester E, De Rocker N, Van Cauwenbergh C, et al Massively parallel sequencing for early molecular diagnosis in Leber congenital amaurosis. Genetics in medicine: official journal of the American College of Medical Genetics 2012, 14:576–585. 22261762 10.1038/gim.2011.51

[12] Fu Q, Wang F, Wang H, Xu F, Zaneveld JE, Ren H, et al Next-generation sequencing-based molecular diagnosis of a Chinese patient cohort with autosomal recessive retinitis pigmentosa. Invest Ophthalmol Vis Sci 2013, 54:4158–4166. doi: 10.1167/iovs.13-11672 23661369 10.1167/iovs.13-11672 PMC3684217

[13] Li H, Durbin R. Fast and accurate short read alignment with Burrows-Wheeler transform. Bioinformatics 2009, 25:1754–1760. doi: 10.1093/bioinformatics/btp324 19451168 10.1093/bioinformatics/btp324 PMC2705234

[14] Li R, Li Y, Fang X, Yang H, Wang J, Kristiansen K, et al SNP detection for massively parallel whole-genome resequencing. Genome research 2009, 19:1124–1132. doi: 10.1101/gr.088013.108 19420381 10.1101/gr.088013.108 PMC2694485

[15] Wei X, Dai Y, Yu P, Qu N, Lan Z, Hong X, et al Targeted next-generation sequencing as a comprehensive test for patients with and female carriers of DMD/BMD: a multi-population diagnostic study. Eur J Hum Genet 2014, 22:110–118. doi: 10.1038/ejhg.2013.82 23756440 10.1038/ejhg.2013.82 PMC3865410

[16] Asan, Xu Y, Jiang H, Tyler-Smith C, Xue Y, Jiang T, et al Comprehensive comparison of three commercial human whole-exome capture platforms. Genome Biol 2011, 12:R95 doi: 10.1186/gb-2011-12-9-r95 21955857 10.1186/gb-2011-12-9-r95 PMC3308058

[17] Badano JL, Kim JC, Hoskins BE, Lewis RA, Ansley SJ, Cutler DJ, et al Heterozygous mutations in BBS1, BBS2 and BBS6 have a potential epistatic effect on Bardet-Biedl patients with two mutations at a second BBS locus. Human molecular genetics 2003, 12:1651–1659. 12837689 10.1093/hmg/ddg188

[18] Katsanis N, Ansley SJ, Badano JL, Eichers ER, Lewis RA, Hoskins BE, et al Triallelic inheritance in Bardet-Biedl syndrome, a Mendelian recessive disorder. Science 2001, 293:2256–2259. doi: 10.1126/science.1063525 11567139 10.1126/science.1063525

[19] Pereiro I, Hoskins BE, Marshall JD, Collin GB, Naggert JK, Pineiro-Gallego T, et al Arrayed primer extension technology simplifies mutation detection in Bardet-Biedl and Alstrom syndrome. European journal of human genetics: EJHG 2011, 19:485–488. doi: 10.1038/ejhg.2010.207 21157496 10.1038/ejhg.2010.207 PMC3060323

[20] Mykytyn K, Nishimura DY, Searby CC, Shastri M, Yen HJ, Beck JS, et al Identification of the gene (BBS1) most commonly involved in Bardet-Biedl syndrome, a complex human obesity syndrome. Nature genetics 2002, 31:435–438. doi: 10.1038/ng935 12118255 10.1038/ng935

[21] Beales PL, Badano JL, Ross AJ, Ansley SJ, Hoskins BE, Kirsten B, et al Genetic interaction of BBS1 mutations with alleles at other BBS loci can result in non-Mendelian Bardet-Biedl syndrome. American journal of human genetics 2003, 72:1187–1199. doi: 10.1086/375178 12677556 10.1086/375178 PMC1180271

[22] Estrada-Cuzcano A, Koenekoop RK, Senechal A, De Baere EB, de Ravel T, Banfi S, et al BBS1 mutations in a wide spectrum of phenotypes ranging from nonsyndromic retinitis pigmentosa to Bardet-Biedl syndrome. Arch Ophthalmol 2012, 130:1425–1432. doi: 10.1001/archophthalmol.2012.2434 23143442 10.1001/archophthalmol.2012.2434

[23] Wang X, Wang H, Sun V, Tuan HF, Keser V, Wang K, et al Comprehensive molecular diagnosis of 179 Leber congenital amaurosis and juvenile retinitis pigmentosa patients by targeted next generation sequencing. J Med Genet 2013. 10.1136/jmedgenet-2013-101558 PMC3932025 23847139

[24] Tosi J, Tsui I, Lima LH, Wang NK, Tsang SH. Case report: autofluorescence imaging and phenotypic variance in a sibling pair with early-onset retinal dystrophy due to defective CRB1 function.Curr Eye Res 2009, 34:395–400. doi: 10.1080/02713680902859639 19401883 10.1080/02713680902859639 PMC2717950

[25] Yzer S, Fishman GA, Racine J, Al-Zuhaibi S, Chakor H, Dorfman A, et al CRB1 heterozygotes with regional retinal dysfunction: implications for genetic testing of Leber congenital amaurosis. Invest Ophth Vis Sci 2006, 47:3736–3744. 10.1167/iovs.05-1637 16936081

[26] Wycisk KA, Zeitz C, Feil S, Wittmer M, Forster U, Neidhardt J, et al Mutation in the auxiliary calcium-channel subunit CACNA2D4 causes autosomal recessive cone dystrophy. Am J Hum Genet 2006, 79:973–977. doi: 10.1086/508944 17033974 10.1086/508944 PMC1698577

[27] Van Den Bossche MJ, Strazisar M, De Bruyne S, Bervoets C, Lenaerts AS, De Zutter S, et al Identification of a CACNA2D4 deletion in late onset bipolar disorder patients and implications for the involvement of voltage-dependent calcium channels in psychiatric disorders. Am J Med Genet B Neuropsychiatr Genet 2012, 159B:465–475. doi: 10.1002/ajmg.b.32053 22488967 10.1002/ajmg.b.32053

[28] Kumar P, Henikoff S, Ng PC. Predicting the effects of coding nonsynonymous variants on protein function using the SIFT algorithm. Nat Protoc 2009, 4(7):1073–81. doi: 10.1038/nprot.2009.86 19561590 10.1038/nprot.2009.86

[29] Adzhubei IA, Schmidt S, Peshkin L, Ramensky VE, Gerasimova A, Bork P, et al A method and server for predicting damaging missense mutations. Nat Methods 2010, 7(4):248–9. doi: 10.1038/nmeth0410-248 20354512 10.1038/nmeth0410-248 PMC2855889

[30] Schwarz JM, Rodelsperger C, Schuelke M, Seelow D. MutationTaster evaluates disease-causing potential of sequence alterations. Nat Methods 2010, 7(8):575–6. doi: 10.1038/nmeth0810-575 20676075 10.1038/nmeth0810-575

[31] Shihab HA, Gough J, Mort M, Cooper DN, Day INM, Gaunt TR. Ranking Non-Synonymous Single Nucleotide Polymorphisms based on Disease Concepts. Human Genomics 2014, 8:11 doi: 10.1186/1479-7364-8-11 24980617 10.1186/1479-7364-8-11 PMC4083756

[32] Pollard KS, Hubisz MJ, Siepel A. Detection of non-neutral substitution rates on mammalian phylogenies. Genome Res 2010, 20(1):110–21. doi: 10.1101/gr.097857.109 19858363 10.1101/gr.097857.109 PMC2798823

[33] Nakamura M, Lin J, Nishiguchi K, Kondo M, Sugita J, Miyake Y. Bietti crystalline corneoretinal dystrophy associated with *CYP4V2* gene mutations. Adv Exp Med Biol 2006, 572:49–53. doi: 10.1007/0-387-32442-9_8 17249554 10.1007/0-387-32442-9_8

[34] Paloma E, Hjelmqvist L, Bayes M, Garcia-Sandoval B, Ayuso C, Balcells S, et al Novel mutations in the *TULP1* gene causing autosomal recessive retinitis pigmentosa. Invest Ophthalmol Vis Sci 2000, 41:656–659. 10711677

[35] Ajmal M, Khan MI, Micheal S, Ahmed W, Shah A, Venselaar H, et al Identification of recurrent and novel mutations in *TULP1* in Pakistani families with early-onset retinitis pigmentosa. Mol Vis 2012, 18:1226–1237. 22665969 PMC3365133

[36] Gu S, Lennon A, Li Y, Lorenz B, Fossarello M, North M, et al Tubby-like protein-1 mutations in autosomal recessive retinitis pigmentosa. Lancet 1998, 351:1103–1104. doi: 10.1016/S0140-6736(05)79384-3 9660588 10.1016/S0140-6736(05)79384-3

[37] Chiang PW, Wang J, Chen Y, Fu Q, Zhong J, Yi X, et al Exome sequencing identifies *NMNAT1* mutations as a cause of Leber congenital amaurosis. Nat Genet 2012, 44:972–974. doi: 10.1038/ng.2370 22842231 10.1038/ng.2370

[38] Neveling K, Collin RW, Gilissen C, van Huet RA, Visser L, Kwint MP, et al Next-generation genetic testing for retinitis pigmentosa. Hum Mutat 2012, 33:963–972. doi: 10.1002/humu.22045 22334370 10.1002/humu.22045 PMC3490376

[39] Xu Y, Guan L, Shen T, Zhang J, Xiao X, Jiang H, et al Mutations of 60 known causative genes in 157 families with retinitis pigmentosa based on exome sequencing. Hum Genet 2014, 133:1255–1271. doi: 10.1007/s00439-014-1460-2 24938718 10.1007/s00439-014-1460-2

[40] Daiger SP, Sullivan LS, Bowne SJ. Genes and mutations causing retinitis pigmentosa. Clin Genet 2013, 84:132–141. doi: 10.1111/cge.12203 23701314 10.1111/cge.12203 PMC3856531

[41] den Hollander AI, Koenekoop RK, Yzer S, Lopez I, Arends ML, Voesenek KE, et al Mutations in the *CEP290* (*NPHP6*) gene are a frequent cause of Leber congenital amaurosis. Am J Hum Genet 2006, 79:556–561. doi: 10.1086/507318 16909394 10.1086/507318 PMC1559533

[42] Perrault I, Delphin N, Hanein S, Gerber S, Dufier JL, Roche O, et al Spectrum of *NPHP6/CEP290* mutations in Leber congenital amaurosis and delineation of the associated phenotype. Hum Mutat 2007, 28:416. 10.1002/humu.9485 17345604

[43] Coppieters F, Van Schil K, Bauwens M, Verdin H, De Jaegher A, Syx D, et al Identity-by-descent-guided mutation analysis and exome sequencing in consanguineous families reveals unusual clinical and molecular findings in retinal dystrophy. Genet Med 2014, 16(9):671–80. doi: 10.1038/gim.2014.24 24625443 10.1038/gim.2014.24

[44] Bellos E, Kumar V, Lin C, Maggi J, Phua ZY, Cheng CY, et al S.cnvCapSeq: detecting copy number variation in long-range targeted resequencing data. Nucleic Acids Res 2014, 42(20):e158 doi: 10.1093/nar/gku849 25228465 10.1093/nar/gku849 PMC4227763

